# Prevalence of Eustachian Tube Dysfunction and Its Associated Factors Among the General Public in Al-Madinah, Saudi Arabia

**DOI:** 10.7759/cureus.33748

**Published:** 2023-01-13

**Authors:** Mansour R Alshamani, Hamza A Alandijani, Omar M Alhussaini, Rayan A Alharbi, Sereen S Almeshaly, Abdulrahman F Alraddadi, Basmah F Zakareya, Ruba D Alrehaili, Faisal A Alkenani, Shahad M Jorob

**Affiliations:** 1 Otology and Neurotology, Ohud Hospital, Almadinah Almunawwarah, SAU; 2 College of Medicine, Taibah University, Almadinah Almunawwarah, SAU; 3 College of Medicine, Alrayan Medical Colleges, Almadinah Almunawwarah, SAU; 4 Medicine, King Abdulaziz University Hospital, Jeddah, SAU

**Keywords:** associated factors, al-madinah, dysfunction, eustachian tube, prevalence

## Abstract

Objectives

To determine the association between patient demographics including gender, age, family history of hearing loss, and eustachian tube dysfunction (ETD) in the Al-Madinah region.

Methods

This is a cross-sectional observational study that was conducted using an online survey in the Al-Madinah region of Saudi Arabia from March 2022 to August 2022. Data were collected using a self-administered questionnaire (ETDQ-7) that was designed based on previous studies and frameworks. It consists of seven items, each with a score ranging from 1 to 7, for a total score of between 7 and 49 points. A total score of 14.5 or above, or a mean domain score of 2.5 or above, is considered abnormal, with higher scores indicating greater severity of symptoms. The analysis was carried out using SPSS v23.

Results

About 380 participants were included in the current study. The mean age of the participants was 29.2 ± 8.7 years old. About 13 (3.4%) of the participants had a history of ETD. The prevalence of ETD among the study population was 41.3%. Gender was significantly associated with the prevalence of ETD (p-value=0.004), with females tending to have the condition more frequently than males. Moreover, the history of ETD was also significant (p=0.001)

Conclusion

A higher prevalence of ETD was found in the current study when compared to international studies, gender and history of ETD were found to be linked with increased prevalence of EDT.

## Introduction

Eustachian tube dysfunction (ETD) is a common disorder that affects the eustachian tube, a narrow tube that connects the middle ear cavity to the nasopharynx and helps to equalize pressure and ventilation in the middle ear [1,2]. ETD can cause poor sound transmission and hearing problems and can lead to other middle ears conditions such as chronic otitis media (COM) and cholesteatoma [3]. ETD can be caused by structural defects in the cleft palate or by extrinsic factors such as upper respiratory tract infections, allergic reactions at the eustachian tube opening, or a combination of both [4].

ETD affects about 1% of the general adult population and about 40% of children experience transient ETD at some point [5,6]. It is seen in 70% of people with middle ear problems such as those who have undergone tympanoplasty for COM or cholesteatoma [7,8]. A study in the United States found that ETD leads to more than two million clinic visits per year among patients aged 20 and above [9]. ETD can cause communication problems, lower productivity, and a poor quality of life. It can also lead to otitis media with effusion (OME), which can cause hearing loss and delay in speech development in children [10].

ETD patients may experience symptoms such as hearing impairment, a feeling of fullness in the ear, or tinnitus. To be diagnosed with ETD, a patient must display these symptoms of pressure imbalance in the affected ear. However, it has been difficult to establish reliable diagnostic procedures and criteria for identifying people with ETD [7]. There are several tools available for assessing eustachian tube function, but they are limited in their use due to the need for expensive equipment and trained personnel, which are only available in specialized health centers [11]. A simple tool such as a self-administered questionnaire, such as the 7-item Eustachian Tube Dysfunction Questionnaire (ETDQ-7), could be a useful tool for clinical practice as it allows patients to evaluate their own symptoms and complaints. The ETDQ-7 is a disease-specific tool for assessing the symptoms of obstructive ETD and its treatment effects [12].

To our knowledge, no study has previously been conducted in the Al-Madinah region of Saudi Arabia to determine the prevalence of ETD in the community. Therefore, we will use the ETDQ-7 instrument to assess the prevalence of ETD in the Al-Madinah region of the Kingdom of Saudi Arabia. Having knowledge about the prevalence of a disease in a population is useful for determining current and future community service needs.

## Materials and methods

This is a cross-sectional observational study that was conducted using an online survey in the Al-Madinah region of Saudi Arabia from March 2022 to August 2022. The survey consisted of a well-structured questionnaire about the prevalence of ETD and its associated factors among the general public in the region. The questionnaire included items on the demographic characteristics of the respondents, factors related to ETD, and its effect on the quality of life of the population in Al-Madinah.

The inclusion criteria for the study included respondents in the region who were 18 years of age or older and who agreed to participate, while those who were under 18 and those who declined to participate were excluded. All participants with allergy, asthma, nasal polyps, tobacco use, ciliary dyskinesia, cystic fibrosis, a past history of ear disease, sensorineural hearing loss, OME, a history of ear infection, prior sinus surgery, exposure to cold/flu symptoms or self-reported allergic rhinitis, exposure to dental overbite, or a confirmed diagnosis of chronic rhinosinusitis were also excluded.

The sample size was calculated using the World Health Organization’s sample size calculator, a confidence level of 95% with a margin of error 5%, and a population size of 2,188,138 from the general public in the Al-Madinah region. The minimum sample size was 380.

This study was conducted on the general population. The questionnaire used in the study was approved by the institutional review board of King Abdul-Aziz University Hospital in Saudi Arabia [13]. The ETDQ-7, a scoring system designed to evaluate the symptoms associated with obstructive ETD, was used in the study [12,14]. It consists of seven items, each with a score ranging from 1 to 7, for a total score of between 7 and 49 points. A total score of 14.5 or above, or an individual mean score of 2.5 or above, is considered abnormal, with higher scores indicating greater severity of symptoms.

The original English version of the ETDQ-7 was translated into Arabic by two independent native Arabic doctors with excellent knowledge of English. The Arabic version was then translated back into English by two independent native English doctors with excellent knowledge of Arabic. The authors compared the back-translated version to the original English version and resolved any differences. This process was followed to ensure that all aspects of the questionnaire were clearly understood by readers. To avoid recall bias, participants were instructed to answer the questions on the ETDQ-7 based on the symptoms they had experienced in the past month.

The study received approval from the Ministry of Health, and participation was voluntary (Registration number: H-03-M-84). Participants were allowed to withhold their consent to participate, and all data from the questionnaire were kept confidential, with only the researchers having access to the participants’ information.

The data were organized in an Excel spreadsheet and analyzed using Statistical Package for the Social Sciences (SPSS) software version 26 (IBM, Armonk, New York, USA). Categorical variables were described using frequency tables, while continuous variables were described using means and standard deviations. Statistical significance was determined using the chi-square test, Fisher’s exact test, and the independent samples t-test. A P-value of less than 0.05 was considered significant.

## Results

In this study, a total of 380 participants were enrolled. The mean age of the participants was 29.2 years, with a standard deviation of 8.7 years. Out of the total participants, 201 (52.9%) were female and 179 (47.1%) were male. Most of the participants (341 or 89.7%) were Saudi Arabian nationals, while 39 (10.3%) were of non-Saudi nationality. Of the participants, 13 (3.4%) had a history of ETD, while 367 (96.6%) had no such history. Five (1.3%) of the participants had a history of hearing loss, while the majority (98.7%) had no history of hearing loss. A family history of hearing loss was present in 100 (26.3%) of the participants, while 280 (73.7%) had no such history. In terms of smoking status, 69 (18.2%) of the participants were smokers, while 311 (81.8%) were non-smokers (Table 1).

**Table 1:** Demographic characteristics of the study participants (n=380)

Variable	Frequency	Percentage
Gender		
Male	179	47.1%
Female	201	52.9%
Nationality		
Saudi	341	89.7%
Non-Saudi	39	10.3%
History of eustachian tube dysfunction		
Yes	13	3.4%
No	367	96.6%
History of hearing loss		
Yes	5	1.3%
No	375	98.7%
Family history of hearing loss		
Yes	100	26.3%
No	280	73.7%
Smoking		
Yes	69	18.2%
No	311	81.8%

In this study, the mean score of the EDTQ was found to be 15.6, with a standard deviation of 8.9. The scores ranged from 7 to 46. The overall prevalence of ETD among the study population was 41.3%. Among the various symptoms included in the questionnaire, the highest average score was recorded for ear symptoms during a cold or sinusitis, while the lowest mean scores were recorded for pressure in the ears and pain in the ears (Table 2).

**Table 2:** Eustachian tube dysfunction-7 (ETD-7)

Question	Score (mean ± SD
Pressure in ears	2.0 ± 1.29
Pain in the ears	2.0 ± 1.30
A feeling that your ears are clogged or under water	2.3 ± 1.62
Ear symptoms when you have a cold or sinusitis	2.6 ± 1.88
Crackling or popping sounds in the ears	2.1 ± 1.73
Ringing in the ears	2.3 ± 1.74
A feeling that your hearing is muffled	2.3 ± 1.71

The analysis found that age was not significantly associated with the prevalence of ETD (p-value=0.481). However, there was a significant association between gender and the prevalence of ETD (p-value=0.004), with females tending to have ETD more frequently than males. Nationality was not significantly associated with the prevalence of ETD (p-value=0.093). A statistically significant association was found between a history of ETD and the prevalence of ETD (p-value=0.001), with patients who had a history of ETD tending to have a higher prevalence of the condition compared to those with no history of ETD. There was no significant association between the prevalence of ETD and a history of hearing loss, family history of hearing loss, or smoking status (p-values=0.164, 0.179, or 0.894, respectively) (Table 3).

**Table 3:** Association between demographic data, past medical history of the participants, and prevalence of eustachian tube dysfunction (ETD) ETD: eustachian tube dysfunction

Parameters	ETD		P-value
	Yes	No	
Age (years) mean ±SD	28.9 ± 8.2	29.5 ± 9.1	0.481
Gender n(%)			
Male	60 (33.5)	119 (66.5)	0.004
Female	97 (48.3)	104 (51.7)	
Nationality n(%)			
Saudi	136 (39.9)	205 (60.1)	0.093
Non-Saudi	21 (53.8)	18 (46.2)	
History of eustachian tube dysfunction n(%)			
Yes	11 (84.6)	2 (15.4)	0.001
No	146 (39.8)	221 (60.2)	
History of hearing loss n(%)			
Yes	4 (80)	1 (20)	0.164
No	153 (40.8)	222 (59.2)	
Family history of hearing loss n(%)			
Yes	47 (47)	53 (53)	0.179
No	110 (39.3)	170 (60.7)	
Smoking n(%)			
Yes	29 (42)	40 (58)	0.894
No	128 (41.2)	183 (58.8)	

## Discussion

The results of this study will help to assess the prevalence of ETD and its associated factors in the community, allowing for a better understanding of the status of the condition and the most common factors associated with it. This information can be used to implement the most suitable interventions to prevent ETD [15].

The prevalence of ETD among the study population was found to be 41.3%, with ear symptoms during a cold or sinusitis having the highest average score on the questionnaire. These results are similar to a study in Jeddah reported by Alshehri et al., who found a prevalence of 42.5%, and also found that ear symptoms during a cold or sinusitis had the highest mean score on the questionnaire [13]. However, the prevalence found in this study is much higher than the United States reported in a study by Shan et al., [16], who found a prevalence of only 4.6%, and also higher than the prevalence of 5.5% reported in a study by Fischer et al. [1]. This higher prevalence in our study may be due to various environmental factors, infections, allergic rhinitis, and other factors.

In this study, a family history of hearing loss was present in 26.3% of the participants, which is similar to the findings of a previous study by Alshehri et al., in which a history of hearing loss was present in more than one-third of the participants [13]. Approximately 18.2% of the participants in this study were smokers. The mean score of the EDTQ was found to be 15.6, which is higher than the score of 14.5 found in a study by Andresen et al. [17].

Age was not found to be significantly associated with the prevalence of ETD, but there was a significant association between gender and the prevalence of the condition, with females tending to have ETD more frequently than males. This finding is consistent with the results of a study by Makibara et al., which also found that females had a higher prevalence of ETD than males [18]. There was no significant association between the prevalence of ETD and a history of hearing loss, family history of hearing loss, or smoking status, which conflicts with the findings of a study by Patel et al., which reported that tobacco smoking was associated with a higher prevalence of EDT [19].

## Conclusions

The current study found a higher prevalence of EDT compared to international studies. History of ETD was also found to be linked with an increased prevalence of EDT. To reduce the risk of developing ETD, efforts should be directed toward educating the community about the most common risk factors for the condition. This could be done through various media outlets, such as the internet and television programs.
